# Supplementary material for: Reduced cloud cover errors in a hybrid AI-climate model through equation discovery and automatic tuning
Source: Sci Rep. 2025 Dec 13;15:43836. doi: 10.1038/s41598-025-29155-3 (PMC12705993; doi:10.1038/s41598-025-29155-3)
Supplement: Supplementary file 1 — Supplementary Information. [file 41598_2025_29155_MOESM1_ESM.pdf]

Supplementary Information

Reduced cloud cover errors in a hybrid AI-climate model through equation discovery and automatic tuning

Arthur Grundner, Tom Beucler, Julien Savre, Axel Lauer, Manuel Schlund, and Veronika Eyring

Supplementary Information

Contents

Supplementary Figures: Figures S1–S10
Supplementary Tables: Tables S1–S4

Supplementary Figures

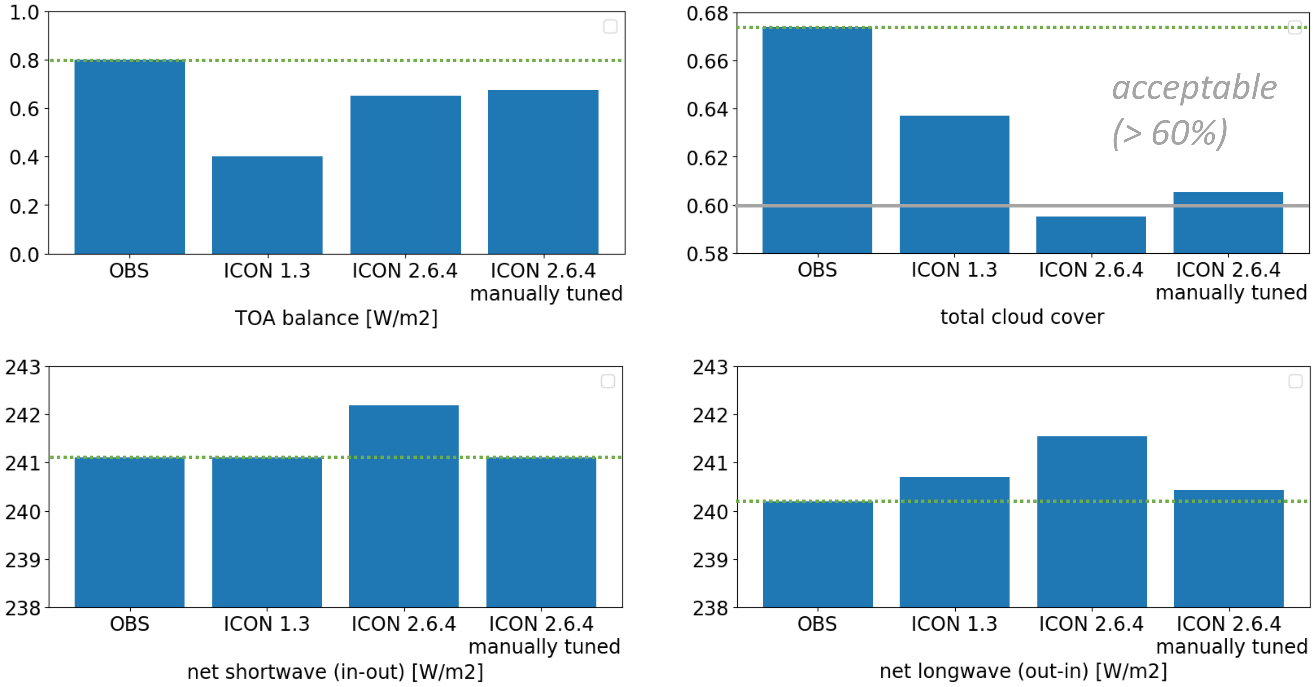

Figure S1: Selection of globally and temporally averaged climate metrics of three 10-year ICON simulations (1980-1989). The ICON-A 2.6.4. simulations used an 80 km horizontal grid, while the original ICON-A 1.3 model simulation ran at an 160 km resolution. The dashed green lines highlight the values from observations, taken from [1]. The lower bound for acceptable total cloud cover values is due to [2].

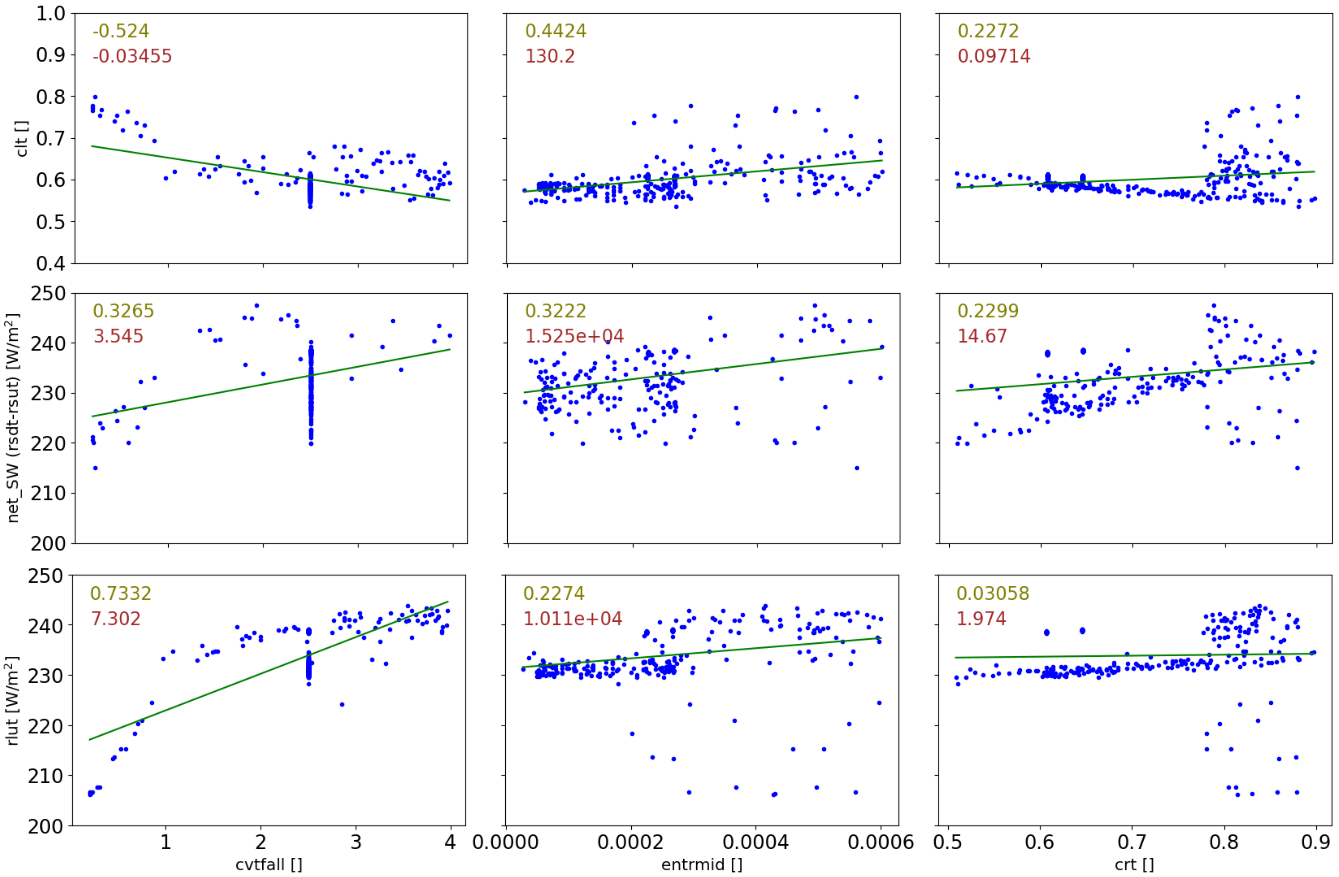

Figure S2: 297 ICON-A simulations with a set of 13 different perturbed parameters (of which only the effects of three are shown). The ordinates describe the global and temporal averages of total cloud cover (clt), the net shortwave and upwelling longwave radiation (rlut) at the top of the atmosphere. The first number reported in each panel are the Pearson correlation coefficients between a given parameter and metric, indicating that *cvtfall*, *entrmid* and *crt* are generally correlated with these three metrics. The second number is the slope of the linear regression line, suggesting how much a parameter should be adjusted to achieve a desired change in a given metric.

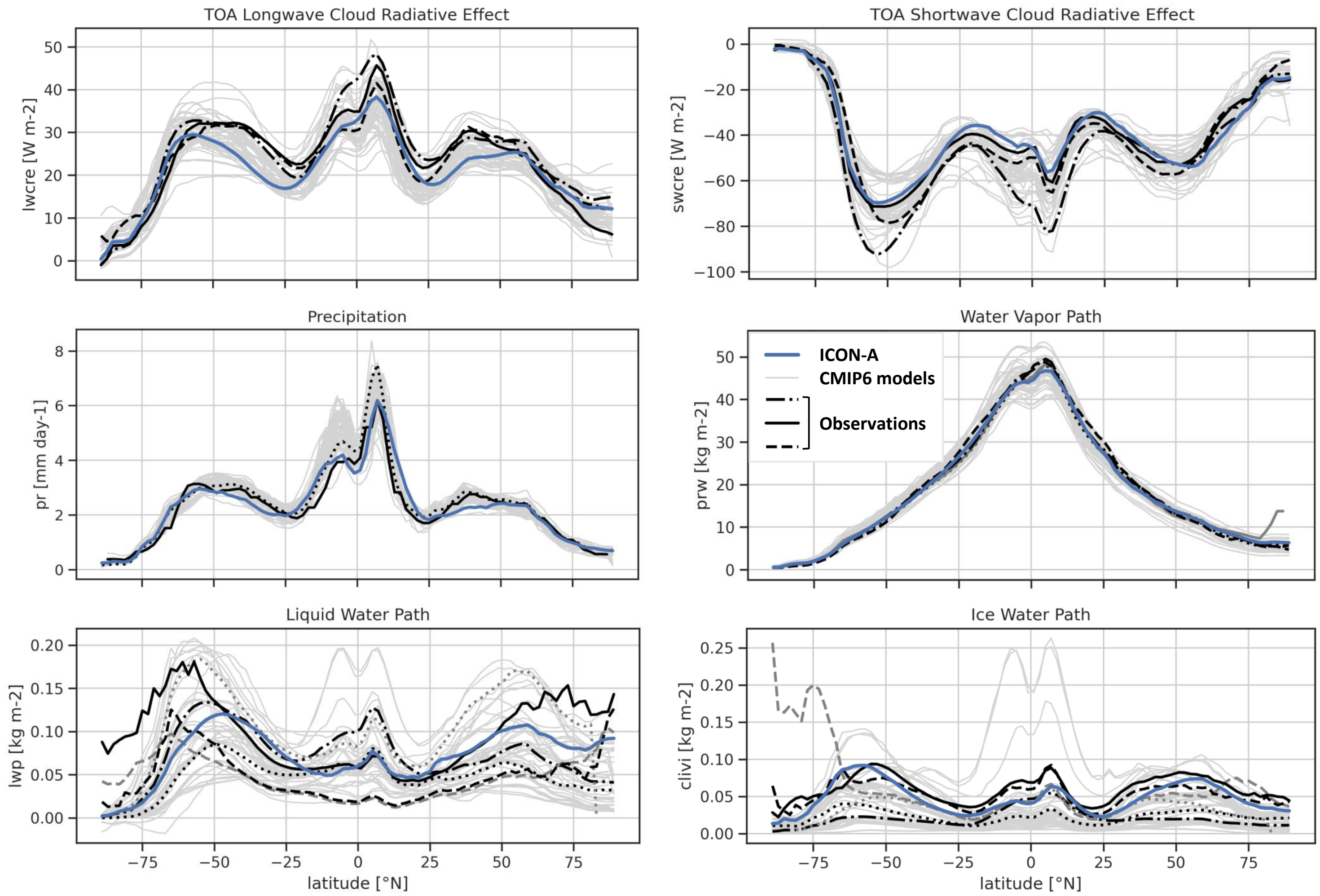

Figure S3: Zonal means of nine important climate variables. The blue lines show zonal means from a 20-year simulation (1979-1999) with the manually tuned ICON-A model, the solid gray lines are based on established CMIP6 model simulations [3] and all other lines correspond to observational sources and reanalysis products.

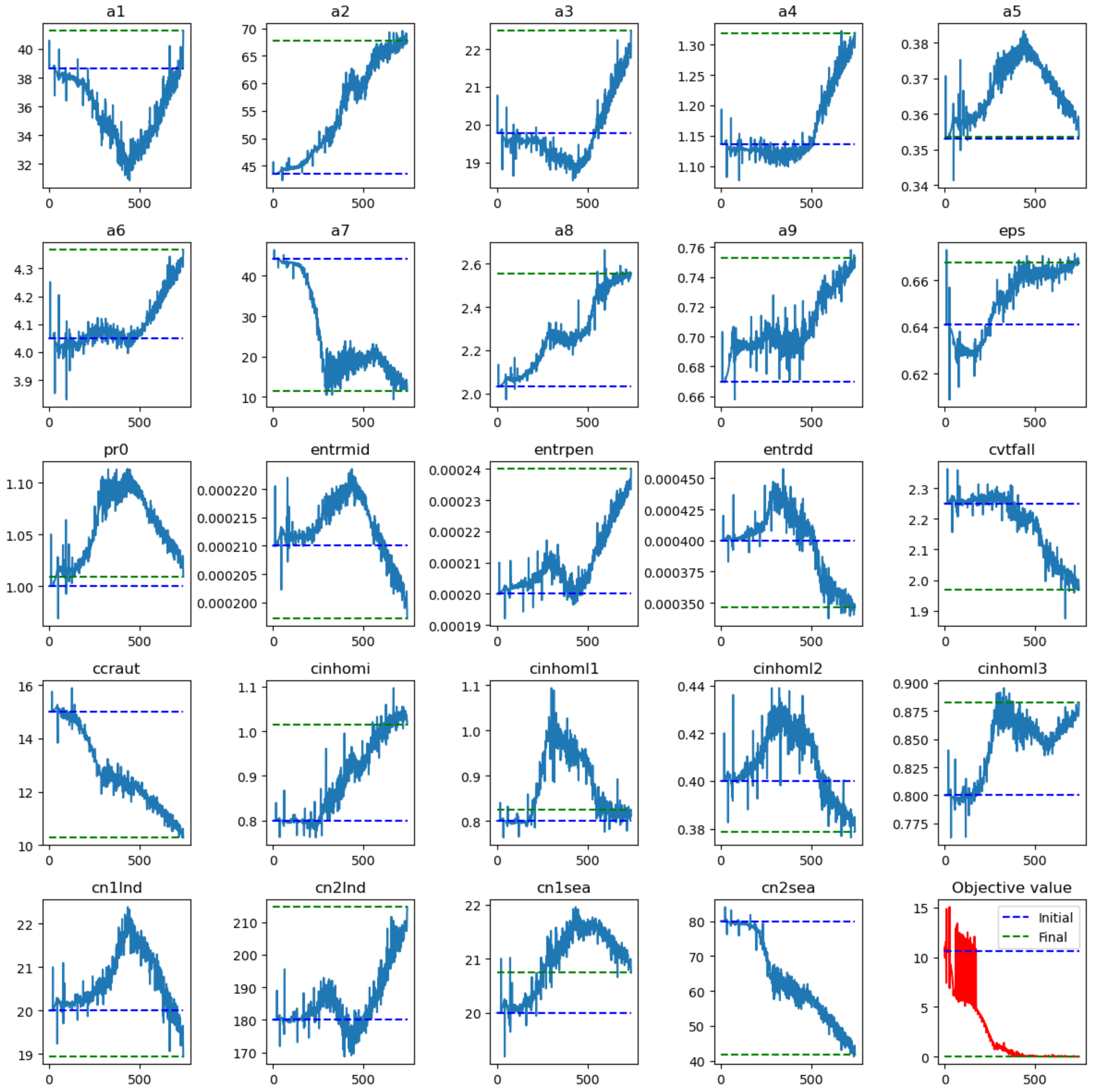

Figure S4: Evolution of tuning parameters (for a description see Table S3) while tuning the ICON-A-MLe model using day-long simulations. The dashed blue line indicates the initial and the dashed green line the final selected parameter values.

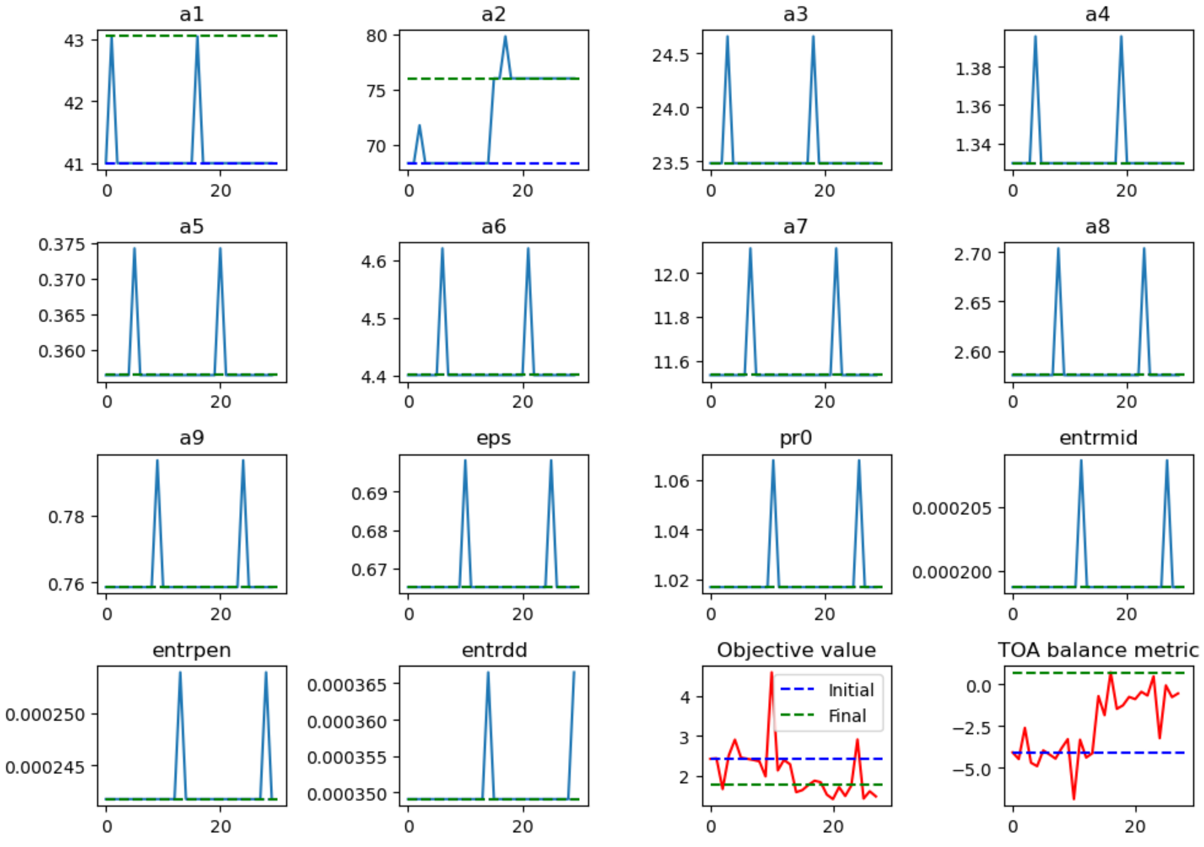

Figure S5: Evolution of tuning parameters (for a description see Table S3) while tuning the ICON-A-MLe model using year-long simulations. The dashed blue line indicates the initial and the dashed green line the final selected parameter values. The parameters that are not shown remain constant.

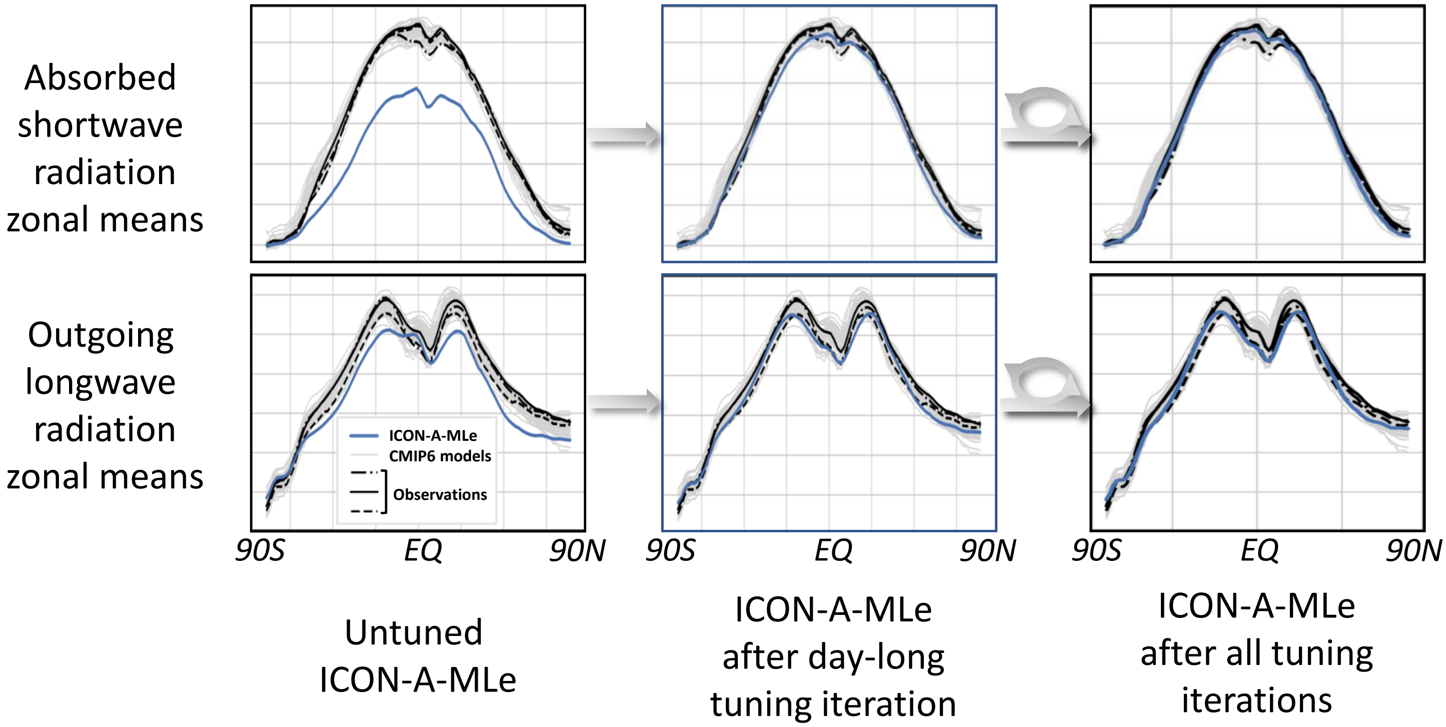

Figure S6: Like Fig. 2, but showing zonal means of the top of the atmosphere longwave and shortwave radiative fluxes.

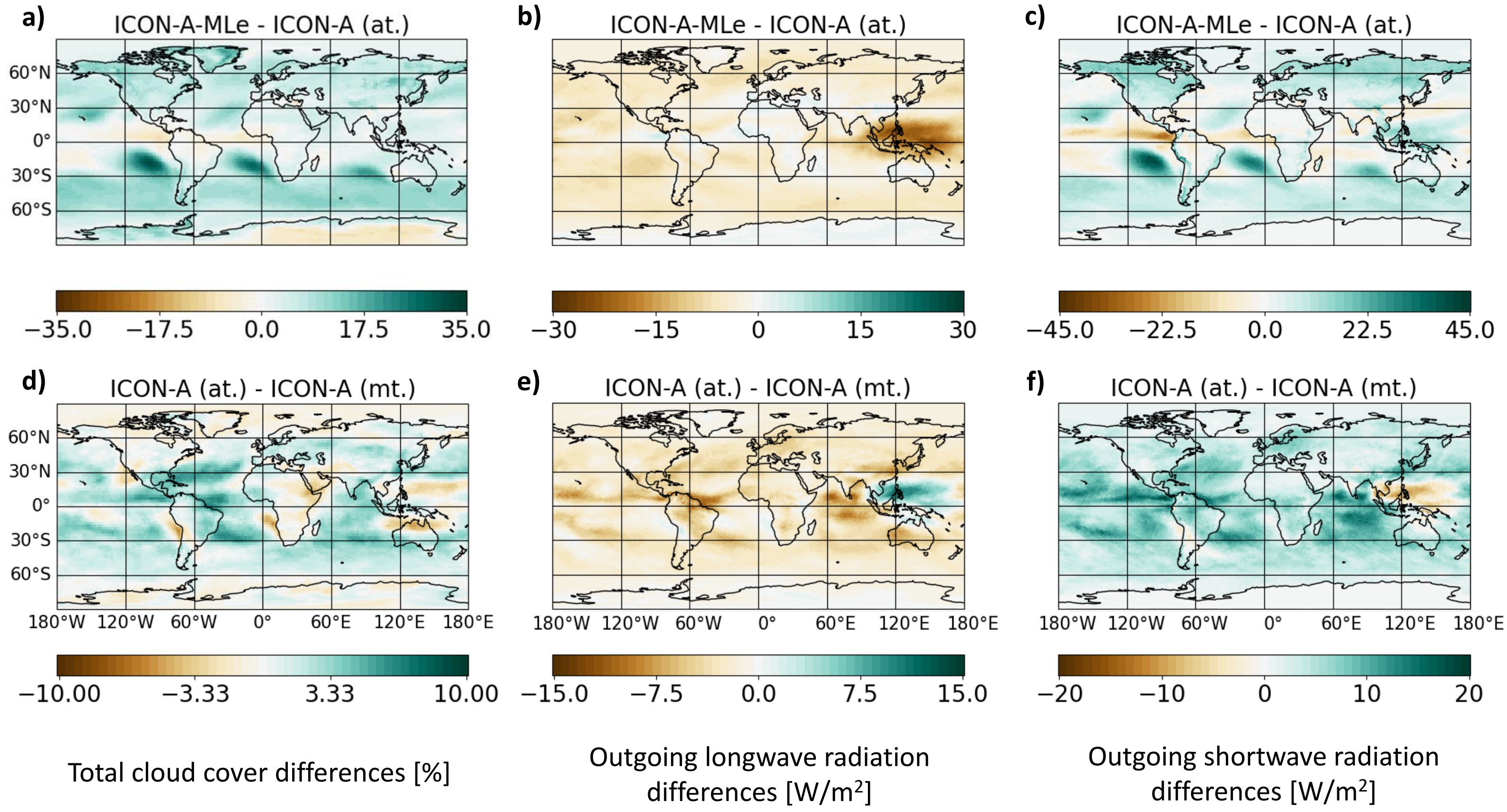

Figure S7: Differences between the panels of each column in Fig. 3, showing bias differences among the automatically tuned (at.) ICON-A-MLe, ICON-A, and manually tuned (mt.) ICON-A models for three key climate metrics over the 1979–1999 period.

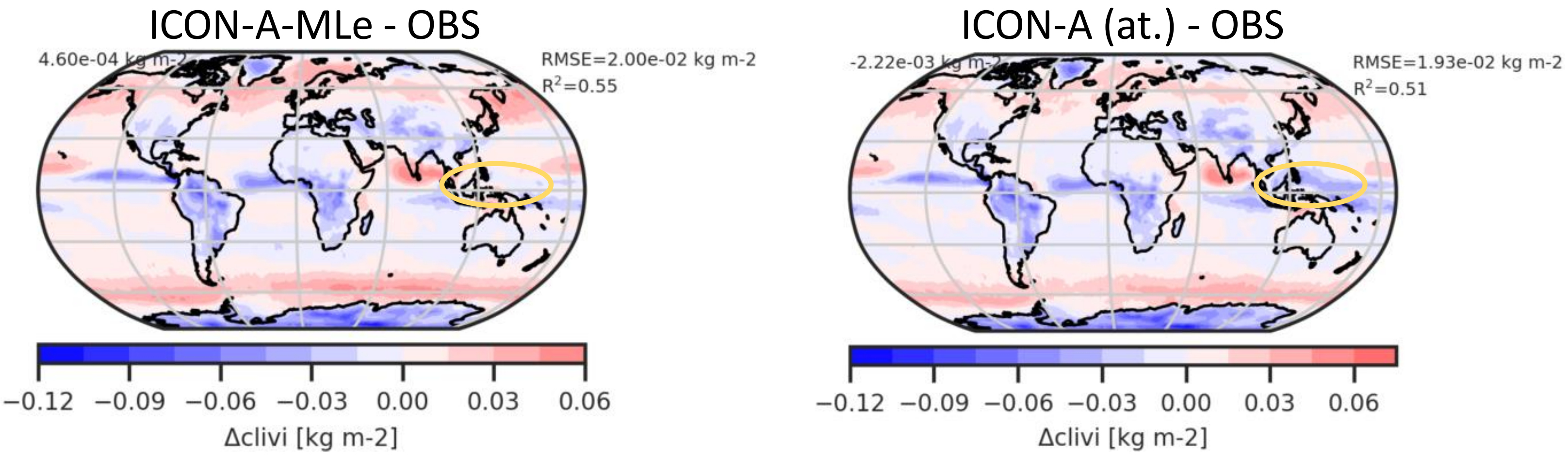

Figure S8: Like Fig. 3, but showing column-integrated cloud ice (ice water path) for the ICON-A-MLe and the automatically tuned ICON-A model simulations. The region highlighted in yellow corresponds to the region around the Philippines in which the ICON-A-MLe model has an increased outgoing longwave radiation bias. The number in the top left provides the difference between the simulated and observational global averages.

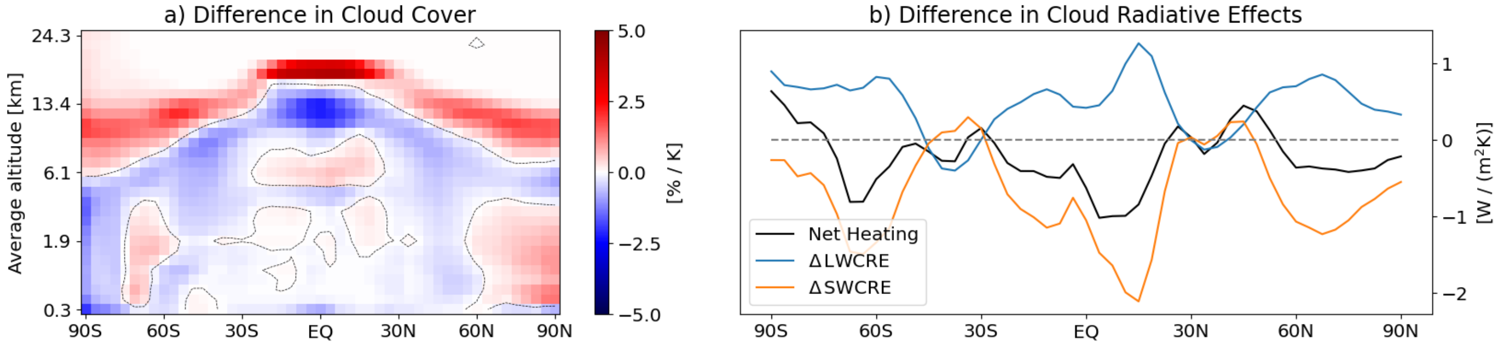

Figure S9: Like Fig. 6, but using the manually tuned ICON-A baseline model to conduct the control and +4K warming scenario simulations.

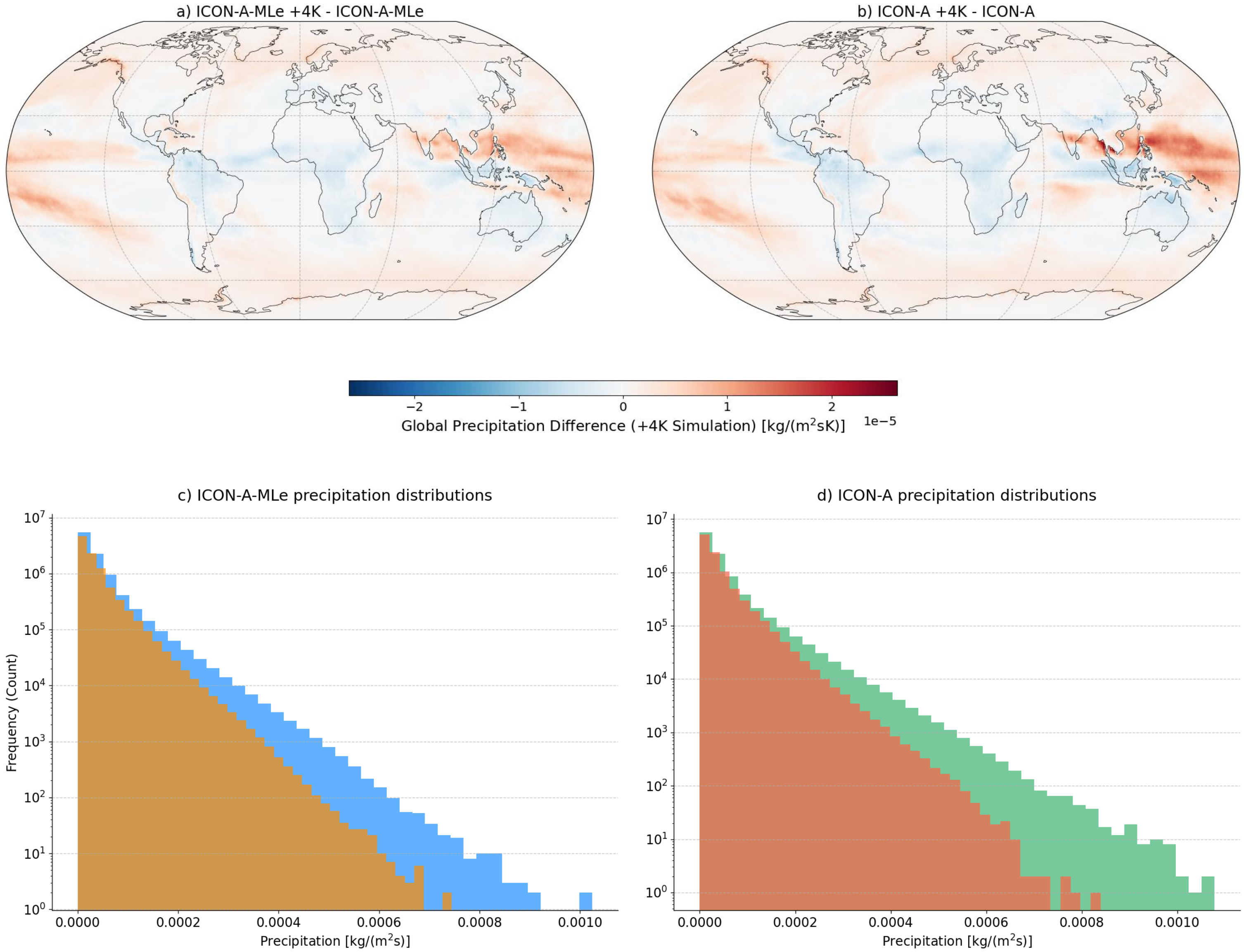

Figure S10: Precipitation metrics. Panels a) and b) illustrate the changes in precipitation per degree of warming relative to a corresponding ICON-A-MLe (at.)/ICON-A (mt.) control simulation without induced heating. Panels c) and d) show histograms of monthly averaged precipitation values. The values are taken from the last 10 years of a 20-year simulation (1979-1999)

Supplementary Tables

Table S1: Evaluation of time-averaged two-dimensional climate variables against observations for our ICON-A-MLe and the native ICON-A simulations. The lower the root mean-squared error (RMSE) and the higher the coefficient of determination (R^2-value) the better. The lowest RMSE values for a given metric across the simulations of Table S1 and S2 are highlighted in bold.

		RMSE (R^2-values)		
Metric	ICON-A-MLe	ICON-A (at.)	ICON-A (mt.)	Reference
Near-Surface Air Temperature	1.22 K (0.99)	1.23 K (0.99)	1.2 K (0.99)	ERA5
Precipitation	1.05 mm/day (0.74)	1.04 mm/day (0.74)	1.09 mm/day (0.73)	GPCP-SG
Ice Water Path	2e-2 kg/m2 (0.55)	1.93e-2 kg/m2 (0.51)	**1.85e-2 kg/m2** (0.57)	MultiOBS1
Liquid Water Path	2.73e-2 kg/m2 (0.56)	2.96e-2 kg/m2 (0.34)	3.38e-2 kg/m2 (0.45)	MultiOBS1
Condensed Water Path	3.34e-2 kg/m2 (0.71)	3.33e-2 kg/m2 (0.67)	3.87e-2 kg/m2 (0.66)	MultiOBS1
Water Vapor Path	2.19 kg/m2 (0.98)	2.06 kg/m2 (0.98)	2.03 kg/m2 (0.98)	MultiOBS2
LW cloud radiative effect	7.22 W/m2 (0.71)	**4.37 W/m2** (0.85)	8.65 W/m2 (0.39)	MultiOBS3
SW cloud radiative effect	**10.19 W/m2** (0.79)	10.29 W/m2 (0.77)	11.43 W/m2 (0.72)	MultiOBS3
Total Cloud Cover	9.87% (0.68)	11.66% (0.58)	12.64% (0.52)	MultiOBS4

MultiOBS1 := {CLARA-AVHRR, CLOUDSAT-L2, ERA5, ESACCI-CLOUD, MERRA2, MODIS}
MultiOBS2 := {ERA5, ESACCI-WATERVAPOUR, ISCCP-FH, MERRA2}
MultiOBS3 := {CERES-EBAF, ESACCI-CLOUD, ISCCP-FH, MERRA2}
MultiOBS4 := {CLARA-AVHRR, ERA5, ESACCI-CLOUD, MERRA2, MODIS, PATMOS-x}

Table S2: Biases of ICON with the data-driven scheme in the auto-tuned ICON-A configuration (ICON-A-MLe*) and of ICON with the native scheme in the auto-tuned ICON-A-MLe configuration (ICON-A*) with subsequent auto-tuning of the cloud cover scheme parameters. The lower the root mean-squared error (RMSE) and the higher the coefficient of determination (R^2-value) the better. The column ML $-$ ¬ML lists ((ICON-A-MLe* $-$ ICON-A) + (ICON-A-MLe $-$ ICON-A*))/2, a measure of how much the data-driven scheme improves a metric.

	RMSE (R^2-values)			
Metric	ICON-A-MLe*	ICON-A*	ML $-$ ¬ML	Reference
Near-Surf. Air Temp.	1.22 K (0.99)	**1.13 K** (0.99)	0.04 K (0.0)	ERA5
Precipitation	**0.98 mm/day** (0.77)	1.12 mm/day (0.7)	-0.07 mm/day (0.04)	GPCP-SG
Ice Water Path	1.88e-2 kg/m2 (0.56)	2.04e-2 kg/m2 (0.5)	-0.06e-2 kg/m2 (0.05)	MultiOBS1
Liquid Water Path	**2.05e-2 kg/m2** (0.73)	4.21e-2 kg/m2 (-0.05)	-1.18e-2 kg/m2 (0.5)	MultiOBS1
Condensed Water Path	**3.15e-2 kg/m2** (0.73)	4.16e-2 kg/m2 (0.52)	-0.51e-2 kg/m2 (0.13)	MultiOBS1
Water Vapor Path	**2.00 kg/m2** (0.98)	2.01 kg/m2 (0.98)	0.01 kg/m2 (0.0)	MultiOBS2
LW cloud radiative effect	5.42 W/m2 (0.81)	6.25 W/m2 (0.75)	1.01 W/m2 (-0.09)	MultiOBS3
SW cloud radiative effect	11.78 W/m2 (0.73)	11.54 W/m2 (0.75)	0.07 W/m2 (0.0)	MultiOBS3
Total Cloud Cover	**9.71%** (0.69)	11.59% (0.54)	-1.77% (0.13)	MultiOBS4

Table S3: Selected ICON-A-MLe(*) tuning parameters and their values. The first 10 listed parameters are part of the data-driven cloud cover equation described in [4].

Parameter	Default values	Tuned values		Short description
		ICON-A-MLe	ICON-A-MLe*	
`a1`	0.444	0.118	0.248	Cloud cover offset
`a2`	1.164	1.234	0.778	Relative humidity sensitivity coefficient
`a3`	-0.015	-0.027	-0.023	Temperature sensitivity coefficient [1/K]
`a4`	4.067	5.65	3.741	Quadratic relative humidity sensitivity coefficient
`a5`	$1.32 \cdot 10^{-3}$	$1.56 \cdot 10^{-3}$	$1.132 \cdot 10^{-3}$	Temperature-relative humidity interaction coefficient [1/K^2]
`a6`	590.01	591.68	421.724	Vertical relative humidity gradient sensitivity scale [m]
`a7`	$2.07 \cdot 10^{-3}$	$2.22 \cdot 10^{-3}$	$5.133 \cdot 10^{-3}$	Vertical relative humidity gradient offset coefficient [1/m]
`a8`	$1.16 \cdot 10^{-6}$	$1.47 \cdot 10^{-6}$	$1.831 \cdot 10^{-6}$	Liquid condensate scaling coefficient [kg/kg]
`a9`	$3.07 \cdot 10^{-7}$	$3.44 \cdot 10^{-7}$	$3.582 \cdot 10^{-7}$	Ice condensate scaling coefficient [kg/kg]
`eps`	1.06	0.615	0.749	Small positive numerical stabilizer
`pr0`	1	1.017	1.059	Neutral limit Prandtl number
`entrmid`	$2.1 \cdot 10^{-4}$	$1.99 \cdot 10^{-4}$	$2.18 \cdot 10^{-4}$	Entrainment rate for midlevel convection
`entrpen`	$2.0 \cdot 10^{-4}$	$2.42 \cdot 10^{-4}$	$2.06 \cdot 10^{-4}$	Entrainment rate for penetrative convection
`entrdd`	$4.0 \cdot 10^{-4}$	$3.49 \cdot 10^{-4}$	$3.98 \cdot 10^{-4}$	Entrainment rate for cumulus downdrafts
`cvtfall`	2.25	1.984	2.186	Coefficient of sedimentation velocity of cloud ice
`ccraut`	15	10.359	16.697	Coefficient of autoconversion of cloud droplets to rain
`cinhomi`	0.8	0.915	0.846	Ice cloud inhomogeneity factor
`cinhoml1`	0.8	0.830	0.865	Liquid cloud inhomogeneity factor, stratiform clouds
`cinhoml2`	0.4	0.382	0.390	Liquid cloud inhomogeneity factor, shallow convection
`cinhoml3`	0.8	0.889	0.832	Liquid cloud inhomogeneity factor, several types of convection
`cn1lnd`	20	19.084	20.987	Cloud droplet num. conc. over land, high altitude [1/cm^3]
`cn2lnd`	180	216.285	177.610	Cloud droplet num. conc. over land, low altitude [1/cm^3]
`cn1sea`	20	20.901	20.206	Cloud droplet num. conc. over sea, high altitude [1/cm^3]
`cn2sea`	80	42.038	81.950	Cloud droplet num. conc. over sea, low altitude [1/cm^3]

Table S4: Selected ICON-A(*) tuning parameters and their values. The first five parameters (*crs, crt, csatsc, nex, cinv*) belong to the native ICON-A cloud cover scheme.

Parameter	Default values	Tuned values		Short description
		ICON-A	ICON-A*	
`crs`	0.968	0.968	0.990	Critical relative humidity at the surface
`crt`	0.8	0.698	0.577	Critical relative humidity at the top
`csatsc`	0.7	0.702	0.703	Minimum saturation for cloud cover below marine inversion
`nex`	2	2.169	2.211	Transition parameter for critical relative humidity profile
`cinv`	0.25	0.249	0.272	Fraction of dry adiabatic lapse rate for inversion top over sea
`pr0`	1	1.059	1.017	Neutral limit Prandtl number
`entrmid`	$2.1 \cdot 10^{-4}$	$2.18 \cdot 10^{-4}$	$1.99 \cdot 10^{-4}$	Entrainment rate for midlevel convection
`entrpen`	$2.0 \cdot 10^{-4}$	$2.06 \cdot 10^{-4}$	$2.42 \cdot 10^{-4}$	Entrainment rate for penetrative convection
`entrdd`	$4.0 \cdot 10^{-4}$	$3.98 \cdot 10^{-4}$	$3.49 \cdot 10^{-4}$	Entrainment rate for cumulus downdrafts
`cvtfall`	2.25	2.186	1.984	Coefficient of sedimentation velocity of cloud ice
`ccraut`	15	16.697	10.359	Coefficient of autoconversion of cloud droplets to rain
`cinhomi`	0.8	0.846	0.915	Ice cloud inhomogeneity factor
`cinhoml1`	0.8	0.865	0.830	Liquid cloud inhomogeneity factor, stratiform clouds
`cinhoml2`	0.4	0.390	0.382	Liquid cloud inhomogeneity factor, shallow convection
`cinhoml3`	0.8	0.832	0.889	Liquid cloud inhomogeneity factor, several types of convection
`cn1lnd`	20	20.987	19.084	Cloud droplet number concentration over land, high altitude [1/cm^3]
`cn2lnd`	180	177.610	216.285	Cloud droplet number concentration over land, low altitude [1/cm^3]
`cn1sea`	20	20.206	20.901	Cloud droplet number concentration over sea, high altitude [1/cm^3]
`cn2sea`	80	81.950	42.038	Cloud droplet number concentration over sea, low altitude [1/cm^3]

References

[1] M. A. Giorgetta et al. “ICON-A, The Atmosphere Component of the ICON Earth System Model: I. Model Description”. In: *Journal of Advances in Modeling Earth Systems* 10.7 (2018), pp. 1638–1662. ISSN: 1942-2466 1942-2466. DOI: 10.1029/2017ms001233.

[2] T. Mauritsen et al. “Tuning the climate of a global model”. In: *Journal of advances in modeling Earth systems* 4.3 (2012).

[3] V. Eyring et al. “Overview of the Coupled Model Intercomparison Project Phase 6 (CMIP6) experimental design and organization”. In: *Geoscientific Model Development* 9.5 (2016), pp. 1937–1958.

[4] A. Grundner, T. Beucler, P. Gentine, and V. Eyring. “Data-driven equation discovery of a cloud cover parameterization”. In: *Journal of Advances in Modeling Earth Systems* 16.3 (2024), e2023MS003763.
